# Biocomputing drug repurposing toward targeted therapies

**DOI:** 10.18632/aging.101135

**Published:** 2016-11-30

**Authors:** Luca Cardone

**Affiliations:** Dept. of Research, Advanced Diagnostic, and Technological Innovation, Regina Elena National Cancer Institute – IRCCS, 00144 Rome, Italy

**Keywords:** oncogenes, PI3K-dependent pathways, gene expression signatures, drugs network, FDA-approved drugs

Targeted inhibition of the oncogenic activation of signalling pathways represents the main goal of drug discovery in oncology. Selective inhibitors of oncogenes such as mutated kinases and phosphatases have been identified mainly through traditional small molecule drug screenings aimed at identifying inhibitors of catalytic activity. However, ever-increasing failure rates, high costs, unsatisfactory safety profile, and limited efficacy are often associated with such traditional drug screenings. Moreover, the escalating costs of anticancer-targeted therapies are generating serious issue of sustainability for all healthcare systems. These inhibitors, even when effective, show paradigms of primary or secondary resistance [1,2]. Thus, additional strategies to identify oncogenic pathway inhibitors should be implemented.

An exciting alternative in drug discovery is to repurpose old, well-known, FDA-approved drugs for novel therapeutic indications, an approach defined as drug repurposing or drug repositioning. Repositioning takes advantage of available pharmacokinetic and toxicity data on existing drugs, limits risks and costs, and thus, accelerates the implementation of new therapies [3].

*In-silico* bio-computational prediction for novel therapeutic indications of FDA-approved drugs can further reduce time and cost efforts necessary for integrating drug repositioning. Our recent paper [4] demonstrated that a specific bio-computational approach [5] could be successfully implemented for repurposing therapeutics able to inhibit oncogenically activated molecular pathways that have a well-established impact on molecular pathogenesis of cancer. This approach is based on modelling specific molecular alterations in cell lines, followed by generating an oncogene-specific gene signature. This molecular signature allowed the inspection of drug network-associated signatures to reposition drugs able to “revert” the oncogenic signature and that could, potentially, act as pathway inhibitors.

As a proof of principle, we focused on oncogenic PI3K-dependent signalling, a molecular pathway frequently driving cancer progression as well as raising resistance to anticancer-targeted therapies. We showed that the implementation of “reverse” oncogenic PI3K-dependent transcriptional signatures combined with the interrogation of drug networks identified inhibitors of PI3K-dependent signalling among FDA-approved compounds. This led, among others, to reposition Niclosamide (Niclo) and Pyrvinium Pamoate (PP), two anthelmintic drugs, as inhibitors of oncogenic PI3K-dependent signalling. Niclo inhibited the phosphory-lation of P70S6K, while PP inhibited the phosphorylation of AKT and P70S6K, which are downstream targets of PI3K. Anthelmintics inhibited oncogenic PI3K-dependent gene expression and showed a cytostatic effect *in vitro* and in mouse mammary gland. Lastly, PP inhibited the growth of breast cancer cells harbouring PI3K mutations. In addition, the inspection of drug communities closed to the PI3K- reverse gene signature predicted off-targets of inhibitors of PI3K pathways and, possibly, side effects in patients treated with targeted therapies.

This computational drug repositioning approach could also complement traditional drug discovery strategy when primary or secondary drug resistance against clinically developed targeted inhibitors emerge. Inhibitors identified via genetic signatures might offer opportunities to target molecular pathways regardless of the specific genetic lesion causing pathway activation. Indeed, genetic signatures can take into account redundancy and heterogeneity of molecular pathway activation that are often the bases of resistance to targeted therapeutics. Consequently, we expect that repurposed drugs by means of gene signatures might be more effective for a larger number of oncogene-dependent cancer phenotypes compared to single kinase inhibitors. Since several studies have demonstrated the potential for using gene expression profiles from cancer cells for the analysis of oncogenic pathways and gene expression profiles reflect patterns of deregulation pathways in cancers [6–7], this approach could be potentially exploited for repositioning drugs targeting deregulated and drug resistance pathways identified by tumour-derived signatures.

In the case of the PI3K pathway, the oncogene-induced signature was sufficient for the computational repurposing of novel pathway inhibitors. This demonstrates one major strengths of this approach, as pathway-specific gene signatures could be generated with high efficacy by genetic manipulation (e.g. by means of somatic KI or Knock-out targeting approaches) for every clinically relevant pathway to be targeted. Thus, the approach could be applied to every genetically targetable oncogene and tumour suppressor mutated in cancer for which pharmacological inhibitors might not be available.

Due to the reasonable costs and high efficacy of techniques for gene editing, such as CRISPR-Cas9, modelling of genetic mutations and generating associated gene signatures could largely be exploited. A part from oncology, it could also be applied for repositioning inhibitors or modulators for pathological molecular pathways deregulated in tissues affected by monogenic mendelian disorders and from which a disease-specific gene expression signature can be derived. Thus, the opportunities coming from the inspection of transcriptional data networks for drug repurposing of targeted therapeutics are just beginning.

**Figure 1:**
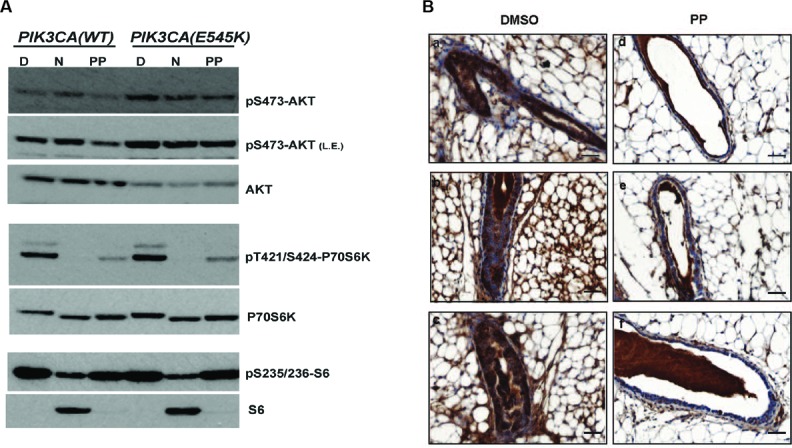
Computational analysis of drug networks and gene expression signatures successfully repurposed new inhibitors of oncogenic PI3K-dependent pathway. (**A**-**B**) Experimental validation of anthelmintics Pyrvinium Pamoate (PP) and Niclosamide (N) as inhibitors of oncogenic PI3K-dependent pathway. (**A**) Wild type human mammary epithelia HME cells (PIK3CA-WT) or isogenic cells carrying the oncogenic *PIK3CA(E545K)* mutation (PIK3CA-E545K) were treated for two hours with DMSO (D) or Niclosamide or Pyrvinium pamoate. Immunoblot analyses of protein lysates showed that targets of PI3K-dependent cascade such as p70S6K and S6 proteins were specifically inhibited after drugs treatments. (**B**) Immunohistochemistry (IHC) analysis of mammary gland sections of female mice treated with DMSO (panels a-c) or PP (panels d-f) and stained with anti phospho-S6 antibodies. Images are representative of different fields of gland sections derived from three different mice (n=3). Images were captured at 40x magnification; scale bars: 30μm.
